# Accelerating and improving radiochromic film calibration by utilizing the dose ratio in photon and proton beams

**DOI:** 10.1002/mp.15828

**Published:** 2022-07-14

**Authors:** Andreas F. Resch, Fatima Padilla Cabal, Milovan Regodic, Wolfgang Lechner, Gerd Heilemann, Peter Kuess, Dietmar Georg, Hugo Palmans

**Affiliations:** ^1^ Division Medical Radiation Physics Department of Radiation Oncology Medical University of Vienna/AKH Wien Vienna Austria; ^2^ MedAustron Ion Therapy Centre Wiener Neustadt Austria; ^3^ Medical Radiation Science National Physical Laboratory Teddington United Kingdom

**Keywords:** radiochromic film, dose calibration, Gafchromic EBT3

## Abstract

**Purpose:**

Radiochromic films are versatile 2D dosimeters with high‐resolution and near tissue equivalence. To assure high precision and accuracy, a time‐consuming calibration process is required. To improve the time efficiency, a novel calibration method utilizing the ratio of the same dose profile measured at different monitor units (MUs) is introduced and tested in a proton and photon beam.

**Methods:**

The calibration procedure employs the dose ratio of film measurements of the same relative profile for different absolute dose values. Hence, the ratio of the dose is constant at any point of the profile, but the ratio of the net optical densities is not constant. The key idea of the method is to optimize the calibration function until the ratio of the calculated doses is constant. The proposed method was tested in the dose range between 0.25–12 and 1–6 Gy in a proton and photon beam, respectively. A radial symmetric profile and a rectangular profile were created, both having a central plateau region of about 3 cm diameter and a dose falloff of about 1.5 cm at larger distances. The dose falloff region was used as input for the optimization method and the central plateau region served as dose reference points. Only the plateau region of the highest dose entered the optimization as an additional objective. The measured data were randomly split into differently sized training and test sets. The optimization was repeated 1000 times with random start value initialization using the same start values for the standard and the gradient method. Finally, a proton plan with four dose levels was created, which were separated spatially, to test the possibility of a full calibration within a single measurement.

**Results:**

Parameter estimation was possible with as low as one dose ratio used for optimization in both the photon and the proton case, yet exhibiting a high sensitivity on the dose level. The root mean squared deviation (RMSD) of the dose was less than 1% when the dose ratio was in the order of 20, whereas the median RMSD of all optimizations was 1.7%. Using four dose levels for optimization resulted in a median RMSD of 1% when randomly selecting the dose levels. Having at least one dose ratio of about 20 included in the optimization considerably improved the RMSD of the calibration function. Using six or eight dose levels reduced the sensitivity on the dose level selection and the median RMSD was 0.8%. A full calibration was possible in a single measurement having four dose levels in one plan but spatially separated.

**Conclusions:**

The number of measurements required to obtain an EBT3 film calibration function could be reduced using the proposed dose ratio method while maintaining the same accuracy as with the standard method.

## 1 INTRODUCTION

Radiochromric films such as Gafchromic EBT3 or EBT‐XD films (Ashland, US) are versatile 2D dosimeters with high resolution and near tissue equivalence resulting in minimal perturbation of the fluence spectrum. The ionizing radiation induces a chemical change in the active material resulting in a change of optical absorption, which is typically quantified using commercial off‐the‐shelf flatbed scanners. The scanner provides a 2D array of pixel values (typically int16 precision), which is sometimes directly used as a measure or converted to optical density or the net optical density, defined as the logarithm of the ratio of the intensities after and before irradiation.^1^ To be able to determine the dose from a film scan, a calibration for a specific film batch to a specific scanner has to be carried out. The quality of the calibration depends on the choice of calibration function, the number of measured dose levels, the number of repetitions, and the number of pixels used for the measurement.^2^, ^3^, ^4^, ^5^

The standard calibration is the most simple but also most robust method: films are exposed to a homogeneous dose distribution at different dose levels. At least 12 dose levels with several repetitions are recommended in one study.^5^ As this is a time‐ and resource intensive process, attempts have been made to find batch‐independent calibrations.^6^, ^7^, ^8^ Yet, none of those methods has been comprehensively validated or found wide‐spread use so far.

Plan‐based calibrations, where films are placed within a dose gradient and using the calculated dose for calibration, allow acquiring data for a large dose range within a single or few measurements.^1^ However, the dose calculation accuracy and misalignment may limit the calibration quality. Furthermore, using a calculated dose as a reference for film calibration may introduce an unwanted correlation if this beam calibration is later on used for verification of the same dose engine.

**FIGURE 1:**
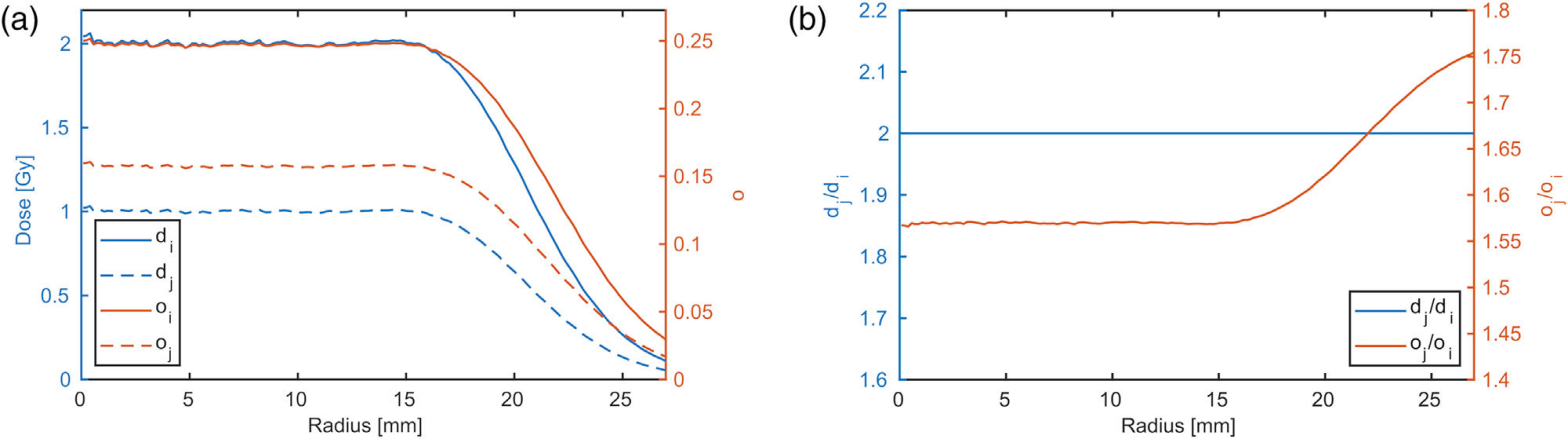
A dose profile *d*(*r*) at two dose levels linearly scaled by constant factors $k_{i}=2$ and $k_{j}=1$ is plotted with blue lines in (a). The corresponding net optical densities, *o*, are calculated applying the calibration function f(d|θ) and are plotted against the right *y*‐axis in red. In (b), the ratios of the two doses and the net optical densities are shown in blue and red, respectively. The dose ratio is constant, whereas the net optical density ratio is not constant

Rosca 2019 proposed a method exploiting a dose gradient without requiring the knowledge of the dose profile.^9^ The same dose profiles are measured with films at different monitor units (MUs) and second to *n‐*th generation dose points can be interpolated from the intercepts. However, a limitation of this method is the propagation of uncertainties with each generation, leading to increased uncertainties with increasing generation. In this study, we propose a similar calibration method exploiting the same dose profile measured at several MUs, but instead of generating new dose points by determining the intercepts, the ratio of the profiles is used. The crucial point is that the ratio of the doses is a constant in that case, but the optical density ratio is not constant as is illustrated in Figure 1. Then, the calibration function is optimized until the calculated dose ratio profile is uniform. In the following sections, the mathematical formalism is derived and the method is experimentally validated in a proton and photon beam. It will be further demonstrated that a dose calibration in a proton beam is feasible within a single measurement of spatially separated dose profiles.

## 2 MATERIALS AND METHODS

### 2.1 Optimization formalism

Let us define the net optical density, *o*, to absorbed dose, *d*, relation as:

(1) $$\begin{array}{cc} d(o) & =f^{-1}\left(o|\theta \right), \end{array}$$

(2) $$\begin{array}{cc} o(d) & =f(d|\theta ), \end{array}$$

given a differentiable function, *f*, with *n* parameters, $\theta =\{\theta_{0},\theta_{1},\text{…},\theta_{n-1}\}$, which is bijective in the relevant dose range. In the literature, *o* is usually plotted as a function of *d*; therefore, we chose to define the function *f* as a function of *d*. Two examples for calibration functions are:

(3) $$\begin{array}{cc} f_{bimol}^{-1} & =\theta_{2}{\left(\frac{o}{\theta_{1}-o}\right)}^{1/\theta_{3}}, \end{array}$$

(4) $$\begin{array}{cc} f_{poly}^{-1} & =\sum_{m=0}^{N}\theta_{m}o^{m}. \end{array}$$

The bimolecular model (Equation (3)) was used to create the artificial data in Figure 1 with parameters from a previous batch.^10^ However, throughout the remainder of the manuscript, a fourth‐order polynomial (Equation (4)) was applied to avoid a bias from the a‐priori knowledge of the start parameters.

We assume a smooth dose profile, d(r), which is normalized to range from 0 to 1, to be dimensionless and a function of the position, *r*. This may be scaled by a constant factor $k_{i}\in Q^{+}$, in our case, the $k_{i}$ corresponds to the “dose level”:

(5) $$\begin{array}{cc} o_{i}\left(r\right) & =f(k_{i}d\left(r\right)|\theta ),\\ k_{i}d\left(r\right) & =f^{-1}\left(o_{i}\left(r\right)|\theta \right). \end{array}$$

To eliminate the unknown d(r) from Equation (5), we form the quotient of two different dose levels, $k_{i}$ and $k_{j}$,

(6) $$\begin{array}{c} \frac{k_{j}}{k_{i}}-\frac{f^{-1}\left(o_{j}\left(r\right)|\theta \right)}{f^{-1}\left(o_{i}\left(r\right)|\theta \right)}=0, \end{array}$$

and we require d,o≠0. This form of the equation could, in principle, be directly translated to a minimum chi‐square expression as it is of the form (measurement value minus model prediction). Although the denominator, $f^{-1}\left(o_{i}\left(r\right)|\theta \right)$ cannot become zero by definition, it may approach zero during parameter optimization. To avoid potential optimization instabilities, we rearrange Equation (6) to

(7) $$\begin{array}{c} \frac{k_{j}f^{-1}\left(o_{i}\left(r\right)|\theta \right)-k_{i}f^{-1}\left(o_{j}\left(r\right)|\theta \right)}{k_{i}f^{-1}\left(o_{i}\left(r\right)|\theta \right)}=0. \end{array}$$

Equation (7) is fulfilled when the numerator is zero. This condition must be true at any point in the profile, and hence, each point in the profile can be treated as an individual data point. We note that this would result in an overdetermined equation system (number of function parameters typically range from 2 to 10, at least one order of magnitude less than data points). For most published calibration functions such as Equation (3), the equation system would be nonlinear. Instead of solving the equation system, we rather determine the function parameters minimizing a cost function χ:

(8) $$\begin{array}{c} \theta =\arg\min_{\theta }\left(\chi \right). \end{array}$$

To derive the cost function, we first define a helper function:

(9) $$\begin{array}{c} h_{i,j,r}^{2}={\left(k_{i}k_{j}\right)}^{-2}{\left(k_{j}f^{-1}\left(o_{i,r}|\theta \right)-k_{i}f^{-1}\left(o_{j,r}|\theta \right)\right)}^{2}, \end{array}$$

which represents the numerator of Equation (7) in discrete notation (replacing (*r*) with index *r*) multiplied with a weighting factor ${\left(k_{i}k_{j}\right)}^{-1}$. The motivation for the weighting factor is to normalize the value of the difference with respect to the dose levels $k_{i,j}$ as both summands in the difference approach $k_{i}k_{j}d_{r}$ when θ approaches the “true” value (use Equation (5)). Equation (9) is the cost of a single data point for one dose ratio. Summing the cost over all positions within a lower and upper boundary $r_{lb,ub}$ and all possible dose ratio combinations makes up the first (of two) parts of the cost function χ:

(10) $$\begin{array}{c} \chi_{a}=\sum_{i=1}^{N_{i}}\sum_{j=i+1}^{N_{i}}\sum_{r=r_{lb}}^{r_{ub}}w_{r}h_{i,j,r}^{2}, \end{array}$$

with the number of dose levels *N* and with an optional weight $w_{r}$ at each position. The optional weighting factor, $w_{r}$, was set to unity throughout this study, but may be useful in certain situations (see Section 4). The double sum over the indices i,j is the combination of all dose levels to one another without double elements. The number of summation operations is $N\left(N-1\right)/2N_{r}$.

Empirically, we found that minimizing $\chi_{a}$ (Equation (10)) does not necessarily result in a unique solution. Therefore, we added an objective, $\chi_{b}$, to the optimization problem and the total cost function is

(11) $$\begin{array}{cc} \chi & =\chi_{a}+\chi_{b}. \end{array}$$

The side objective

(12) $$\begin{array}{c} \chi_{b}=\sum_{i}^{N_{d}}w_{b}{\left(f^{-1}\left(o_{i}|\theta \right)-d_{i}\right)}^{2}, \end{array}$$

minimizes the quadratic difference between a measured dose, $d_{i}$, and the calibration function value of all measured doses $N_{d}$. Equation (12) is the cost function for calibration in spatially homogeneous dose distributions, which is the standard (or reference) method at our and many other institutes. In this study, only one dose (the highest dose) was used as additional objective to demonstrate the feasibility of our method.

The partial derivatives are :

(13) $$\begin{array}{cc} & \frac{\partial }{\partial \theta_{m}}\chi \left(\theta \right)=\frac{\partial }{\partial \theta_{m}}\chi_{a}\left(\theta \right)\\ & \;+\sum_{i}2w_{b}\left(f^{-1}\left(o_{i}|\theta \right)-d_{i}\right)\frac{\partial }{\partial \theta_{m}}f^{-1}\left(o_{i}|\theta \right),\;\mathrm{with}\\ & \frac{\partial }{\partial \theta_{m}}\chi_{a}\left(\theta \right)=\sum_{i=1}^{N_{i}}\sum_{j=i+1}^{N_{i}}\sum_{r=r_{lb}}^{r_{ub}}2w_{r}h_{i,j,r}{\left(k_{i}k_{j}\right)}^{-1}\\ & \;\left(k_{j}\frac{\partial }{\partial \theta_{m}}f^{-1}\left(o_{i,r}|\theta \right)-k_{i}\frac{\partial }{\partial \theta_{m}}f^{-1}\left(o_{j,r}|\theta \right)\right), \end{array}$$

which are required for many optimization algorithms.

With Equations (11) and (13), the minimization to determine the function parameters as defined in Equation (8) can be carried out using as few as one dose ratio (two dose levels). Film measurements are typically repeated several times (three in our case) to compensate for the inherent uncertainties of the films and scanner. Repeated measurements of the same dose level were here treated as individual measurement building the ratio of all measurements to each other without the measurements of the same dose level. This means that the sum in Equation (11) goes over i=1 to 3*N* for three repetitions. The number of ratios used for optimization is then 9(N)(N−1)/2.

The value of the first summand in Equation (11) is Equation (10), which depends on the number of measurements $N_{i}$ and number of positions $N_{r}$ between the lower and upper boundary of *r*. The second summand only depends on the goodness of the fit and consists of $N_{d}$ data points, which is here only one data point. To ensure a balance in the cost function the factors, $w_{r}$ and $w_{b}$ need to be normalized. In this work, we normalized on the number of summation operations choosing $w_{r}=1$ and $w_{b}=w_{s}\;N_{r}\;9N_{i}\left(N_{i}-1\right)/2$, with an arbitrary weighting factor $w_{s}=10$.

Minimizing Equation (11) to obtain θ is referred to as the dose ratio method in the following. Using Equation (12) will be referred to as the standard method.

### 2.2 Measurements

Three measurement sessions were carried out, one using a photon beam (X‐1) and two using proton beams. An overview can be found in Table 1. The first proton measurements (P‐1) consisted of 12 (one dose level per plan) and the second (P‐2) 3 measurements (each with four dose levels per plan). In addition to the different spatial arrangement of the dose profiles, the proton plans differed conceptually in the choice of dose levels. The first measurement was carried out to find an optimal combination of dose levels, which were defined as calibration dose levels before conducting the experiment and analysis.

**TABLE 1:** Overview of the three measurement sessions

Measurement session	X‐1	P‐1	P‐2
Beam	photon	proton	proton
Modality	sequential	sequential	simultaneous
Profile	axial	radial	radial
Number of measurements	6	12	3
Dose levels per plan	1	1	4
Number of dose levels	6	12	12
Dose range [Gy]	1 ‐ 6	0.25 ‐ 12	0.5 ‐ 12
Dose level distribution	uniform dose	uniform opt. density	irregular

All dose profiles exhibited a central (3 cm) plateau region and a dose gradient region to allow to use the same measurement data for the standard and the ratio calibration method. The central plateau region also served as verification of the obtained calibration curves. The profiles were radial (proton) or axial (photon) symmetric to minimize sensitivity to misalignment.

The EBT3 film scanning protocol is similar to earlier studies,^10^, ^11^, ^12^ which can be briefly summarized as follows. Films were scanned individually at the center of a flatbed scanner in transmission mode, portrait orientation, and approximately 48 h before and after exposure. The net optical density was then calculated as the logarithm with base 10 of the mean pixel intensity before irradiation and the pixel value after irradiation of the red channel. The resolution was set to 300 dots per inch corresponding to a scanned pixel side length of 0.08 mm. As the experiments were carried out at two different sites, two different film batches and scanners available on‐site were used to avoid environmental disturbance of the films during transport. Proton and photon beam measurements were carried out at the MedAustron Ion Therapy facility (Wiener Neustadt, Austria)^13^ and General Hospital Vienna (Vienna, Austria) using the respective film dosimetry protocol with different dose levels and ranges for calibration. The lot number of the films used for proton beams was #03122003 and for photons #05062004. Two different EPSON (EPSON GmbH, Meerbusch, Germany) flatbed scanners were used: a DIN A3 sized Epson 11000 XL for protons and an A4 sized Epson Perfection V700 for the photon measurements. The EBT3 sheets (20.3 × 25.4 cm) were cut into quadratic pieces with 6.7 cm side length.

#### 2.2.1 Protons

A treatment plan (TP) was optimized with Matlab to generate a radial symmetric dose profile using pencil beam scanning. The underlying dose profile for the optimization was generated with Gate/Geant4 Monte Carlo simulations using a detailed description of the beamline, which was validated in earlier studies.^14^, ^15^ The dose profile at 2 cm water equivalent depth of a single 179.2 MeV proton beam was simulated and scored as a function of radius. The number of particles of the individual PBs in the TP was optimized to result in a dose profile that is homogeneous up to approximately 15 mm radius and falls to zero dose toward larger radii. A pixel‐wise cost function (squared deviation) was optimized, where the goal was set to a uniform dose in the central region and a linearly decreasing dose in the approximately 80–20% dose falloff region. The calculated dose distribution of the resulting TP is plotted in Figure 1. The initial number of particles of this TP (P‐1) was scaled linearly to irradiate 12 dose levels, such that the dose in the central plateau reached 0.25, 0.50, 0.75, 1.00, 1.50, 2.00 2.5, 3.0, 5.00 7.5, 10.0, and 12.00 Gy. For each dose level, a stack of three EBT3 films was placed in‐between RW3 plates such that the water equivalent depth of the active area of the films was centered at 19.7, 20.1, and 20.5 mm. The reference dose at the center of the field was verified with a Roos chamber (TM34001, PTW, Freiburg, Germany) having a circular active area with a 15.6 mm diameter. Note that the beam monitors at the experimental site were calibrated in terms of number of particles rather than MUs.^16^, ^17^

An additional set of three measurements (P‐2) was carried out 6 months later . A TP defining identical fields (the same as in P‐1) but with different dose levels and spatially separated were delivered in one run. The centers of the fields were spaced 12 cm apart minimizing the scatter contribution from one field to another and being within the maximum field size of 20 × 20 cm. To quantify the distortion of the dose distribution due to in‐scattering, the TP was recalculated with Gate/Geant4. The dose of one field without neighboring fields was calculated and the TP with all four fields.

#### 2.2.2 Photons

A Versa HD linear accelerator (Elekta AB, Stockholm, Sweden) was used to produce a 4× 4 cm field using a nominal beam energy of 6 MV and a source‐to‐surface distance of 90 cm. EBT3 films were positioned one by one at 10 cm depth in a 30×30×30 cm Gammex solid water phantom (Sun Nuclear, Melbourne, FL, USA). Absorbed dose to water was determined in the same phantom setup using a PTW 30006 Farmer chamber (PTW, Freiburg, Germany) in a 10× 10 cm field following TRS‐483.^18^, ^19^ The phantom dose conversion factor was previously determined to be unity with an uncertainty of 0.1%. The number of MUs to deliver 1–6 Gy in 1 Gy steps in the 4× 4 cm field was calculated based on the determination of absorbed dose in the 10× 10 cm field considering the field output factor of the 4× 4 cm field of 0.879. Experiments were carried out in service mode manually defining leaf and jaw position. The output factor was measured with a Semiflex TM31010 (PTW, Germany), which does not require a correction for small field effects for this field size.^18^

### 2.3 Implementation

#### 2.3.1 Converting 2D to 1D optical density profiles

All measured 2D profiles were converted to 1D line profiles exploiting their symmetry. The photon profiles were averaged along one of the two symmetry axis, but limited to a distance of 1 cm from the axis, to remain within a homogeneous dose region.

To convert the 2D pixel grid of the proton measurements into a 1D radial dose profile, the center of the profile was determined by minimizing the standard deviation, $\sigma_{o}^{r}$, of the optical density along a constant radius. As the dose profile was known a‐priori, the radius where the dose reaches 50% of the central dose plateau was chosen. First, $\sigma_{o}^{r}$ was determined for center positions on a rough grid around the geometric center of the image. Second, $\sigma_{o}^{r}$ was calculated on each point of a fine grid centered around the minimum of the first iteration. The minimum of the second iteration was chosen as the center of the circular field and optical densities were rebinned as a function of the radius.

#### 2.3.2 Parameter estimation

To determine the fit parameters, an unconstrained minimization function, *fminunc()* using the *trust‐region* option in MatlabR2020a (The MathWorks, USA), was used to minimize the objective function equation (11) using the objective gradient equation (13). The dose and optical density of the central region r≤7.8 mm entered the optimization as an additional objective. The initial start values for the parameters were selected randomly from a normal distribution. Both the net optical density and the dose of the training data were normalized to range from 0 to 1.

In addition to the newly proposed method, the standard method (Equation (12)), where only the dose and net optical density within a homogeneous dose region was used to obtain the fit parameters, was evaluated for P‐2. The homogeneous central region served as input for the standard optimization method, whereas only the gradient region was used for the ratio method.

#### 2.3.3 Parameter verification

The remaining data sets not used for the fit procedure were used for verification, where only the homogeneous dose region in the center of the field was selected. The relative dose deviations as a function of dose and the root mean square deviation (RMSD) were calculated for each calibration curve serving as an assessment parameter of the goodness of the fit.

### 2.4 Analysis

In the results section, we will first present all measured 2D profiles converted to radial or line profiles. Each data set was later on split into a group serving as training data for the optimization and a second group serving for verification referred to as test data. The dose levels were randomly selected to reduce human sampling bias for the X‐1 and P‐1 data.

In the second step, the accuracy of the determined calibration function in terms of dose was determined over the entire dose range for some example cases using the measurements in the photon and proton beam.

In the next step, the number of dose levels required to determine a calibration function was investigated for the P‐2 data set. Therefore, the quality of the calibration function (in terms of RMSD) as a function of the number of dose levels used for optimization was systematically investigated using a subset of 2, 4, 6, or 8 of the 12 dose levels for optimization. The distribution of the RMSD of the 10^4^ individual optimizations (randomly selecting the initial start values and the dose level in each run) is visualized using violin plots, which allows to estimate both the quality and stability of the fit from the median and the variance, respectively. Further, to find the optimal dose levels for optimization, the special case of a single dose ratio (two dose levels) was investigated. For more dose levels, where the number of combinations increases rapidly, patterns were investigated manually finding similarities in fits resulting in RMSDs less than the median.

Finally, a calibration was carried out using a single measurement of four dose levels separated in space (P‐2), where the dose levels for training were defined before the measurement. Monte Carlo simulations of the entire dose plan are shown to estimate the mutual dose disturbance of the four fields.

## 3 RESULTS

### 3.1 Measured profiles

**FIGURE 2:**
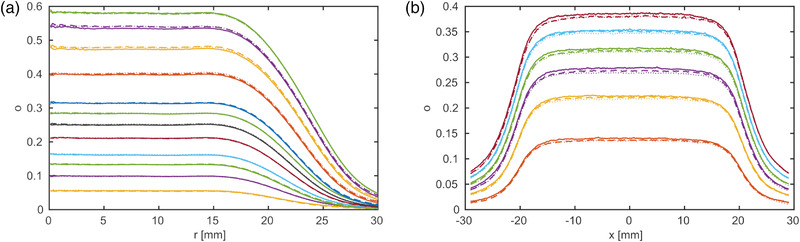
Measured net optical densities of the proton (a) and photon beam (b) as a function of the radius, ***r***, or lateral position, ***x***, averaged over the area. The graphs show different dose levels, which is represented with different colors. Each dose level was measured three times, which is represented with solid, dashed, and dotted lines

The measured net optical densities converted to radial and line profiles are shown in Figure 2 for the proton and photon measurements (X‐1 and P‐1). The profiles confirm a homogeneous dose plateau region in the center of the films and a dose falloff from about 15–30 mm distance. Due to the finite size of the film pieces (67× 67 mm) and to keep a safety margin of at least 5 mm to the edge, only data from 15 to 27 mm were selected for parameter optimization. Including data from the plateau region did not add relevant information, but slowed down the optimization and was therefore not used.

The three film measurement repetitions can be clearly identified in the plateau region in the photon measurements in Figure 2(b), which points to a poorer repeatability of the film/scanner combination compared to the proton measurements. Ionization chamber measurements did not show any problem in repeatability (standard deviation was 0.1%).

### 3.2 Photon beam

**FIGURE 3:**
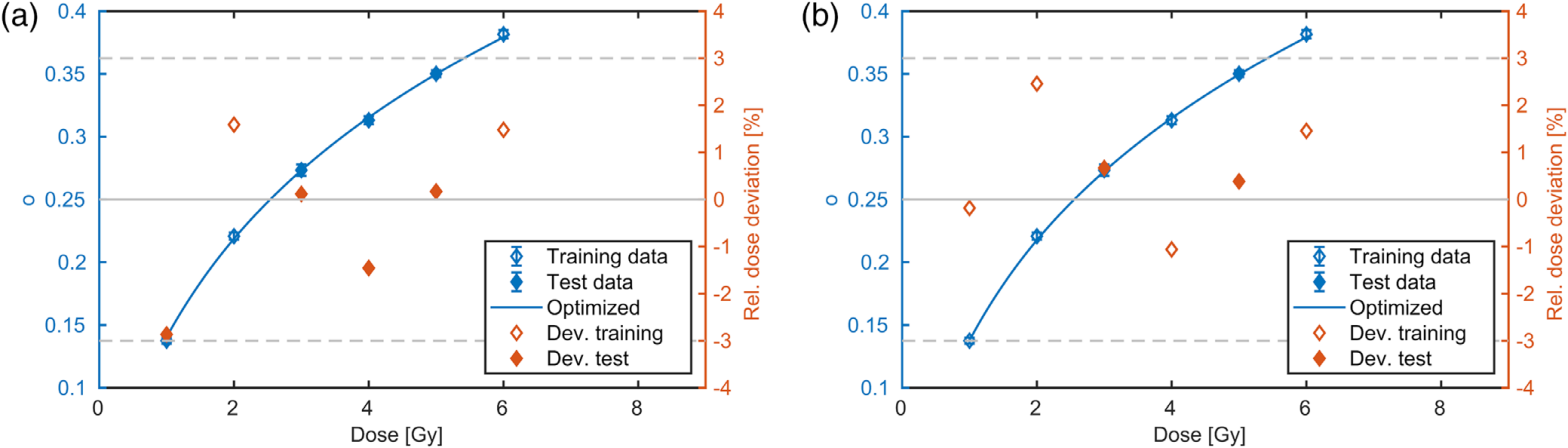
Two examples of calibration curves using four of six photon data sets for parameter estimation using different dose levels and start parameters. The net optical density is represented in blue (left *y*‐axis) as a function of dose. The fit result is represented with a line in the valid dose region. The relative dose deviation of the fit result to the measurement is shown in red (right *y*‐axis). Measurements used for training or testing are represented with open or filled diamonds, respectively. The 3% dose deviation is indicated by dashed horizontal lines

Example calibration curves obtained from the photon measurements using the proposed ratio method, Equation (11), are shown in Figure 3, demonstrating the applicability of the proposed optimization strategy. Two or four out of the six measured profiles were used for optimization. The residuals show the deviation of the dose derived from the mean optical density of the central plateau region applying the calibration function and the dose measured with the ionization chamber. Dose deviations were less than 3%, which was about the order of the variance observed from the three repetitions.

### 3.3 Proton beam

**FIGURE 4:**
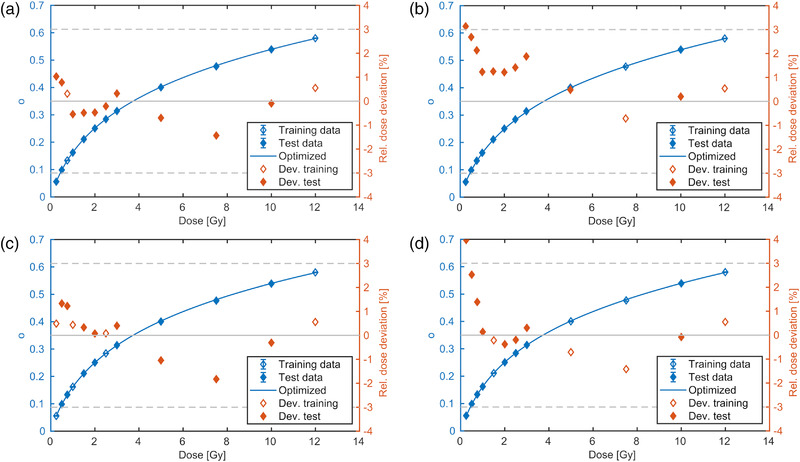
Measured (points) and optimized (line) net optical density to dose relations of the proton measurement P‐1. The data sets selected for optimization are represented with open diamonds, whereas the ones for testing are marked with filled diamonds. Two and four out of 12 data sets were used for fitting in the upper and lower panel, respectively. Different dose levels and start parameters were used in the optimization of the calibration curve resulting in relatively low or high residuals in the left and right column, respectively

Four examples of determined calibration curves for the proton beam (P‐1) using the proposed ratio method are shown in Figure 4. The dose profiles with a dose plateau value of 0.75 and 12 Gy (ratio 16) were used for optimization in Figure 4(a), whereas dose levels 8 and 12 Gy (ratio 1.5) was used in Figure 4(b). Four dose levels were used for optimization in Figures 4(c) and (d). The calibration curves resulted in dose residuals of less than 3% with exception of the 0.25 Gy point, which exceeded this threshold in some runs. It shall be noted that a fit with as low as two dose levels was sufficient to result in a calibration curve with less than ±3% deviation. The different distribution of the deviations in the two runs with different dose levels and start parameters indicate a systematic dose level and start value dependence.

### 3.4 Determination of optimal dose levels and repeatability

**FIGURE 5:**
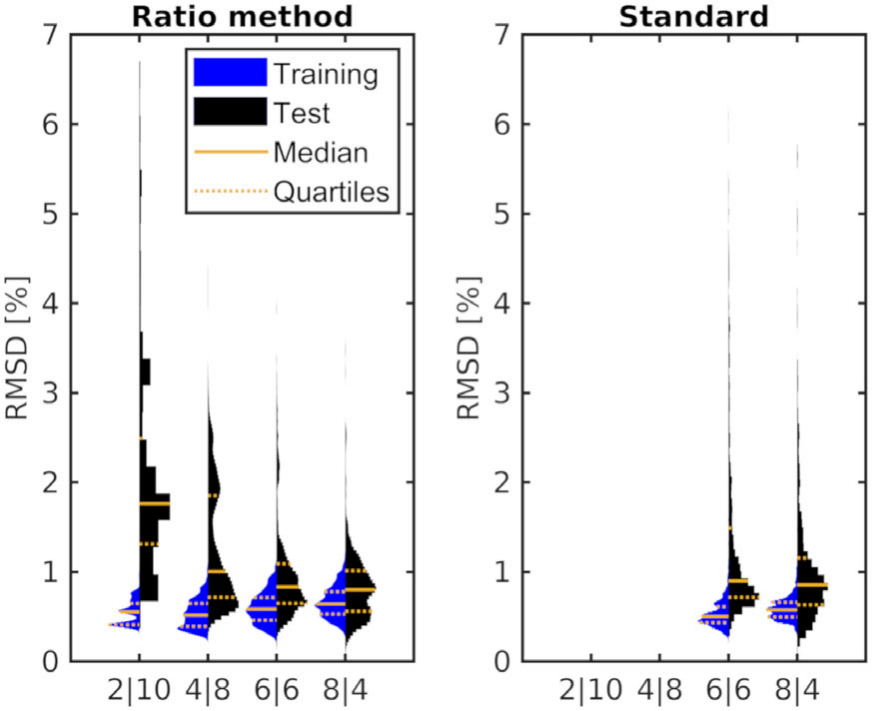
Root mean square (RMSD) of 1000 independent fits splitting the 12 proton data sets at random into a subset of 2, 4, 6, or 8 dose levels for training (blue) and the remaining (10, 8, 6, 4) for testing (black). The results of the ratio and standard methods are shown in the left and right graph, respectively. The RMSDs of the standard method were only evaluated if the number of training data exceeded the number of free parameters. The 25, 50, and 75 percentiles are indicated with orange lines

Figure 5 shows the violin plot of the RMSD (of the dose residuals) in 1000 independent runs using randomly selected dose levels and random start values for the optimization. Using only two or four dose levels for calibration resulted in a median RMSD of 1.7% and 1.0% in the test data set. Increasing the number of training data to 6 or 8 reduced the median RMSD to 0.8% showing a moderate decrease from 6 (0.83%) to 8 (0.80%) measurements. Thus, using six training data appeared to be sufficient to determine the calibration function when the dose levels were randomly selected.

The standard method using the homogeneous dose region for fitting resulted in a median RMSD of 0.9% (see Figure 5), which was higher compared to the ratio method (0.8%) using the same number of training data. The dose ratio optimization did not only result in a lower median RMSD, but also a narrower distribution, which suggests that the ratio method based fit was more stable than the standard approach for the measurements in this study. The RMSD was lower in the training compared to the test data set in both methods. This was more pronounced for the standard method and points to overfitting.

**FIGURE 6:**
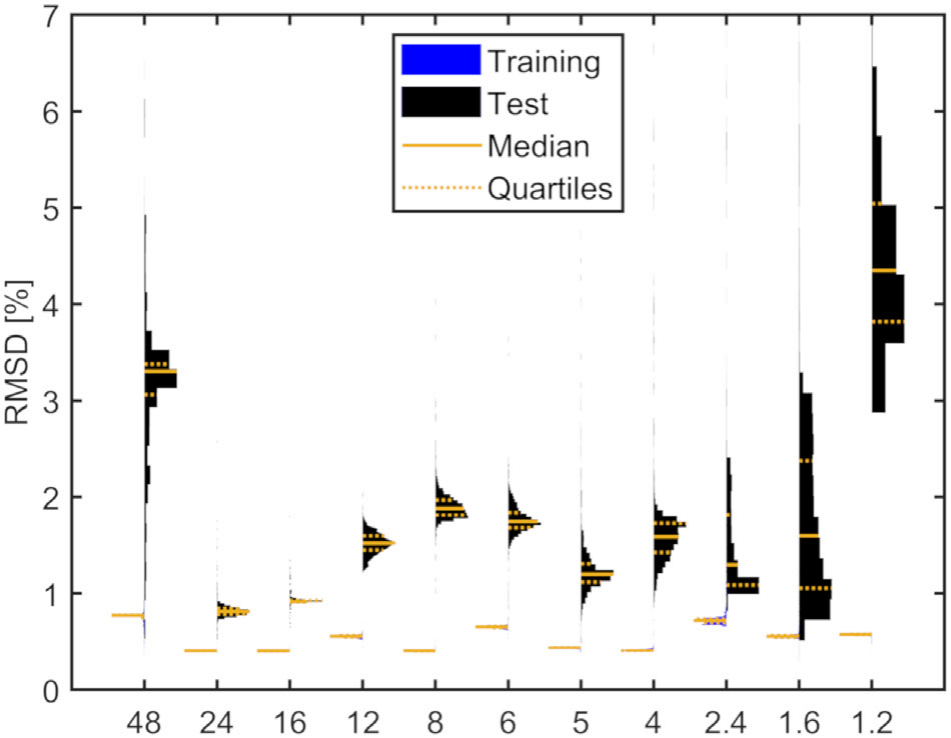
The RMSD as a function of the dose ratio for the special case of only using two training data sets (one ratio) out of the 12 proton data sets (P‐1). The 25, 50, and 75 percentiles are indicated with orange lines

Using less than six training data resulted in a strong dependence on the dose levels used for optimization, which ended in wide and multivariate RMSD distributions. The dose‐level dependency can be illustrated for the special case of only two dose levels: the violin plots of the RMSD are plotted as a function of the training dose ratio in Figure 6. Highest RMSDs occurred when the lowest (1.2) or highest dose ratio (48) was used for training. Lowest RMSDs in the test data were obtained for the training dose ratios ranging from 16 to 24 (corresponding to 0.75 and 0.5 Gy). The fit quality dependence on the selected dose levels is complex in the situations using four or more dose levels for fitting. We found that having at least one training dose level measured at less or equal 1 Gy yielded best calibration curves reducing the median RMSD to 0.8% in the four dose level fit situation.

### 3.5 Calibration in a single measurement

**FIGURE 7:**
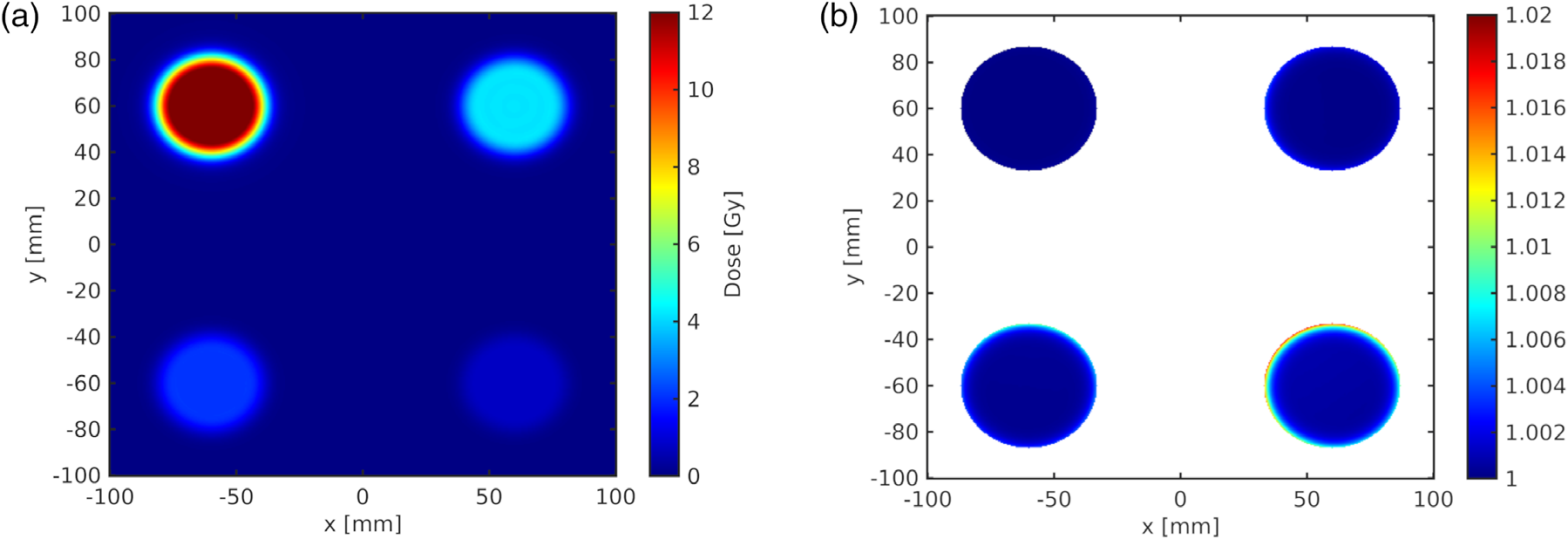
The calculated proton (P‐2) dose distribution of four dose levels on one plane is shown in (a) and the ratio of the perturbed to the unperturbed dose is shown in (b) up to a constant radius that corresponds to the 5% dose level (white outside the threshold). The dose at the center of the field was 4, 12, 2, and 0.75 Gy in the quadrants I (upper right) to IV (bottom right), respectively

Figure 7 shows the MC calculated dose distribution of the four radial profiles in one TP allowing for a simultaneous measurement of four dose levels (P‐2). The mutual influence of the fields appears at the low dose area, but reaches only up to about 1% for the lowest dose profile (0.75 Gy) at the lateral cutoff value (the radius of 5% of the central dose value). Consequently, a distortion of the calibration due to the scatter contributions from the other fields could be excluded.

**FIGURE 8:**
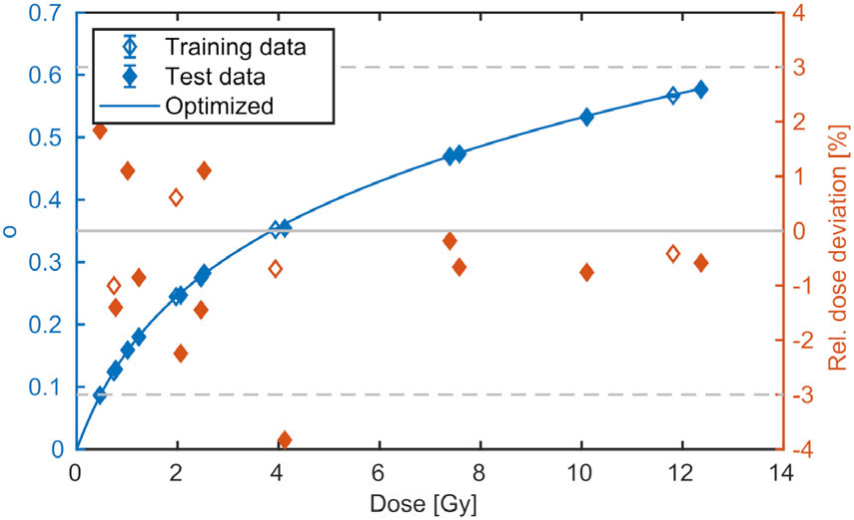
Calibration function using the four field maps with protons (P‐2)

Using the four dose levels in one measurement for calibration resulted in a similar calibration quality (see Figure 8) as in the previous case of consecutive measurements of a single central field. The dose residuals were within ±3% with one outlier (−3.8%) and the RMSD was 0.7%.

## 4 DISCUSSION

In this study, a film calibration method was proposed and verified within a non‐radial and radial symmetric dose distribution. The radial symmetric distributions were chosen as they offer independence of translational and rotational misalignment within the measurement plane. Accurate alignment is essential in the non‐symmetric case, but may be corrected applying image postprocessing. Our results demonstrated that calibration was feasible in both cases without correcting for misalignment. However, there is no restriction to line profiles and a 2D array could also be used. Therefore, one could use the dose profile, which should be determined with the films as calibration profile, which could allow for a sheet specific calibration. The absolute dose entering the optimization problem as side objective can be retrieved from any region of interest within the same dose profile, but can also come from a separate measurement under reference conditions. All dose profiles in this study were monotonically decreasing in the region used for optimization, but this is not required. More complex dose distributions such as patient TPs, step profiles, or chess pattern like profiles could even further improve the dose ratio method.

The proposed ratio method is a variant from the Rosca 2019 method, which is based on interceptions. The advantages of the ratio method lie in the low requirements on the underlying dose profile and simple formal description, whereas the interception method is more intuitive and optimization is carried out similar to the standard method. Both methods allowed to reduce the measurement time for film calibration. Which model performs better may depend on the underlying dose profile and the dose levels used as both models seem to benefit from a non‐uniform dose level distribution.

In the standard method, one measurement gives one data point in the fitting problem. Therefore, about eight or more measurements are necessary to perform a reliable fit with a calibration function with five free parameters. In the dose ratio method, each pixel within a dose gradient is one data point allowing to reduce the number of measurements to as low as two, which resulted in more than 2000 data points in the optimization. We believe that this large number of data points, which corresponds to an overdetermined optimization problem, may explain several beneficial observations. First, the optimization was stable and rather insensitive to the choice of start parameters (with the restriction of allowing only physically meaningful start parameters, i.e., parameters resulting in positive doses). Second, oscillations, which are a common problem in polynomial fitting, were not observed. However, there is probably a cross‐correlation of oscillations with the choice of start parameters, which were sampled from a distribution centered around zero to start with small values. It will be repeated here that the number of measured dose levels in the standard method must exceed the number of free parameters, which is in practice often restricted by time. The insufficient number of calibration points (as low as 3–5) used even in advanced dosimetry applications has been discussed in a recent study.^20^ Although the number of dose levels in the dose ratio method could be reduced to as low as two, at least four may be recommended to increase reliability and result still in a relevant time reduction.

As this method is based on the dose ratio as obtained from the steep dose gradients, any geometric changes to the profile will deteriorate the quality. One of such factors is the physical stress caused by cutting the films, which results in a bending of the films and hence a variation of the air gap distance between film and scanner glass.^21^ This effect can be minimized using tempered glass on top of the films during scanning or cutting the films a few days before use.^21^, ^22^ A further systematic bias may be caused by scanner artifacts such as non‐homogeneous lateral response and polarization effects. In this study, they were not corrected, but the effects were minimized by using small film pieces (≈3 cm radius) and placing them at the center of the scanner where those corrections are minimal.^20^, ^23^, ^24^ It would be possible to use larger films, if scanner corrections are applied, which would offer shallower depth dose gradients and consequently more pixels for each “calibration point.” Scanner artifacts are typically more problematic toward the edges of the field of view making DIN A4 scanners (as used in the photon experiments) more susceptible to those uncertainties. However, the high variance in the net optical density in the photon film measurements must origin either in the specific EBT3 batch or the Epson Perfection V700 scanner. Uncertainties on the determined dose increase at low optical densities; therefore, the dose calibration was considered valid between the lowest and highest measured values. This excluded the potential extrapolation to zero optical density and required the highest dose value to be part in the dose ratio.

The EBT3 film response to absorbed dose is sensitive to the beam quality. The beam quality should therefore be sufficiently constant within the dose profile to have a negligible impact on the film response. This is typically the case for measurements perpendicular to the beam direction in a proton or high energy (MV) photon beam. The red channel was used because most beam quality studies in proton beam therapy are related to the red channel.

To use the proposed method Equation (11) needs to be implemented and minimized with an optimization algorithm of the users' choice. The side objective in Equation (11) was the standard method using only one dose value in this study to demonstrate the functionality of the method. However, in practice, there is no need to exclude the other dose values. Here, the dose gradient method was applied only to the gradient region. This may be recommended to avoid including a plethora of points with redundant information, slowing down the optimization and noise may become the driving force. In the case of the radial dose distribution, the variance is decreasing with increasing radius due to the increasing number of pixels contributing to $o_{r}$. The variance could be included in the weighting factor $w_{r}$.

The factor, $w_{s}$, which weights the cost of the absolute dose in the total cost function, was here set to 10. Increasing the weight by several orders of magnitude forced the fit function to go through the highest dose point resulting in a zero dose residual at that point. Lowering the weighting factor resulted in increasing dose residuals. Therefore, it may be recommended to start off with a high value and reduce it if necessary.

The ratio method was tested with EBT3 films in this study. There is no apparent reason, which prohibits the use of this method with other types of radiochromic films such as EBT‐XD. In fact, the ratio method should work with any 2D detector with high resolution and a sufficiently uniform response.

## 5 CONCLUSIONS

This work demonstrated that an alternative film calibration method using information from the same dose profile delivered at different MU levels can provide an at least equally accurate and less time consuming method for both photon and proton beams. Although it was possible to determine a net optical density to absorbed dose calibration using as few as two dose levels (one dose ratio), we recommend using at least four to reduce sensitivity on the chosen dose levels. The further potential of the ratio method lies in using larger films and more complex dose distributions.

## CONFLICT OF INTEREST

The authors have no conflicts to disclose.
